# *FOXR2* activation is not exclusive of CNS neuroblastoma

**DOI:** 10.1093/neuonc/noaf076

**Published:** 2025-04-15

**Authors:** Alexa N Siskar, Emily Hanzlik, Maria F Cardenas, Mohammad K Eldomery, Soniya Pinto, Christopher L Tinkle, Qunyu Zhang, Xiaoyu Li, Tong Lin, Sandeep K Dhanda, Gerald Reis, Daphne Li, Ravi Raghavan, Alexander Vortmeyer, Matthias A Karajannis, Giles W Robinson, Arzu Onar-Thomas, Patrick R Blackburn, David A Wheeler, Jason Chiang

**Affiliations:** Department of Pathology, St. Jude Children’s Research Hospital, Memphis, TN, USA; Department of Computational Biology, St. Jude Children’s Research Hospital, Memphis, TN, USA; Department of Pediatric Medicine, St. Jude Children’s Research Hospital, Memphis, TN, USA; Department of Computational Biology, St. Jude Children’s Research Hospital, Memphis, TN, USA; Department of Pathology, St. Jude Children’s Research Hospital, Memphis, TN, USA; Department of Radiology, St. Jude Children’s Research Hospital, Memphis, TN, USA; Department of Radiation Oncology, St. Jude Children’s Research Hospital, Memphis, TN, USA; Department of Pathology, St. Jude Children’s Research Hospital, Memphis, TN, USA; Department of Pathology, St. Jude Children’s Research Hospital, Memphis, TN, USA; Department of Biostatistics, St. Jude Children’s Research Hospital, Memphis, TN, USA; Department of Oncology, St. Jude Children’s Research Hospital, Memphis, TN, USA; Memorial Healthcare System, Hollywood, FL, USA; Pediatric Neurosurgery, Advocate Children’s Hospital, Park Ridge, IL, USA; Department of Pathology, Loma Linda University Medical Center, Loma Linda, CA, USA; Department of Pathology, University of Mississippi Medical Center, Jackson, MS, USA; Department of Pediatrics, Memorial Sloan Kettering Cancer Center, New York, NY, USA; Department of Oncology, St. Jude Children’s Research Hospital, Memphis, TN, USA; Department of Biostatistics, St. Jude Children’s Research Hospital, Memphis, TN, USA; Department of Pathology, St. Jude Children’s Research Hospital, Memphis, TN, USA; Department of Computational Biology, St. Jude Children’s Research Hospital, Memphis, TN, USA; Department of Pathology, St. Jude Children’s Research Hospital, Memphis, TN, USA

**Keywords:** alternative promoter, CNS tumors with *FOXR2* overexpression, LINE-1 retrotransposition, patient outcomes, promoter donation and enhancer hijacking

## Abstract

**Background:**

*FOXR2* activation is regarded as pathognomonic for CNS neuroblastoma (NB). However, a comprehensive understanding of the landscape for CNS tumors exhibiting *FOXR2* activation is lacking.

**Methods:**

Histopathologic, molecular, imaging, and clinical data of 42 CNS tumors with *FOXR2* overexpression identified through screening institutional datasets and published institutional cases were analyzed.

**Results:**

Among the 42 tumors, 21 (50.0%) were high-grade gliomas (HGGs), and 18 (42.9%) were embryonal tumors. The HGGs included ten H3 K27M-mutant diffuse midline gliomas (DMGs) and eight radiation-associated tumors. The embryonal tumors included 11 CNS NBs and six pineoblastomas (PBs). *FOXR2* expression was similar between CNS NB and other tumor types (*P* = 0.82). HGGs with *FOXR2* overexpression, unlike NBs and PBs, displayed diverse concomitant genetic alterations. The most common mechanisms of *FOXR2* activation involved structural alterations causing promoter donation and enhancer hijacking from active genes essential for brain development, followed by alternative promoter activation or truncated LINE-1 retrotransposition. The preferential activation mechanism varied by tumor type. All but two aberrant *FOXR2* transcripts incorporated non-canonical, non-coding exons. Gene set enrichment analysis demonstrated shared downstream effects of *FOXR2* activation at the epigenome and transcriptome levels across tumor types. DMGs and PBs with *FOXR2* overexpression were aggressive, with 0% 2-year overall survival, whereas CNS NBs responded well to combined chemotherapy and radiation.

**Conclusions:**

CNS tumors with *FOXR2* overexpression manifest significant histological, molecular, imaging, and clinical diversity. While HGGs and PBs with *FOXR2* overexpression demonstrated inferior prognosis, CNS NBs showed favorable outcomes. Integrating histologic and molecular diagnostic approaches is imperative for accurate prognostication and optimal therapeutic decision-making.

**Key Points**

1. CNS tumors with *FOXR2* overexpression manifest significant histological, molecular, and clinical diversity.
2. *FOXR2*-activated CNS neuroblastoma showed favorable outcomes.
3. Integrating histologic and molecular information is imperative for accurate diagnosis.

**Importance of the Study**

This study on CNS tumors with *FOXR2* overexpression offers a substantial advancement in understanding these tumors’ diverse histopathologic, molecular, and clinical features. While previous research predominantly focused on *FOXR2*’s role in CNS neuroblastomas, this study broadens the landscape by demonstrating its activation across various CNS tumors, including high-grade gliomas and other CNS embryonal tumors. By employing an integrated approach that combines transcriptomic, genomic, epigenomic, and clinical data, the research uncovers distinct activation mechanisms and associated survival outcomes specific to each tumor type. It highlights the critical importance of combining histologic and molecular diagnostics for accurate prognostication and personalized treatment planning. The findings also pave the way for future targeted therapies aimed at *FOXR2*’s downstream molecular pathways.

FOXR2 (Forkhead Box R2) is a transcription factor belonging to a large family of highly conserved FOX proteins known for their roles in regulating gene expression and a wide array of cellular functions.^1^ Normally expressed only in the testis, *FOXR2* has been identified as an epigenetically activated oncogene in many cancers.^2^ In the CNS, *FOXR2* activation is considered the pathognomonic molecular signature of CNS neuroblastoma (NB)^3–5^ and has been demonstrated to be causative in developing CNS embryonal tumors.^6^ However, more recent studies indicate that *FOXR2* may be implicated in a broader range of CNS tumors. These studies have implicated *FOXR2* in subtypes of pineoblastoma (PB),^7^ medulloblastoma (MB),^8^ and high-grade glioma (HGG).^2,9^ However, a comprehensive understanding of the landscape for CNS tumors exhibiting *FOXR2* activation is lacking, and the exact underlying mechanisms of *FOXR2* activation in specific brain tumor types have yet to be elucidated. Previous studies have demonstrated the association of elevated *FOXR2* expression with poor prognosis in some cancers.^10–12^ However, whether *FOXR2* overexpression has implications for therapeutic responses and clinical outcomes in specific CNS tumor types remains unknown. Moreover, since *FOXR2*-activated CNS NB is rare, collecting as much information as possible about their clinicopathologic profiles is essential for gaining further insights into their treatment response and prognosis.

We hypothesized that CNS tumors with *FOXR2* overexpression exhibit diverse histopathologic, molecular, and clinical features. To understand the landscape of CNS tumors with *FOXR2* overexpression, we screened the institutional whole-transcriptome gene expression (RNA-seq) datasets to identify tumors with *FOXR2* overexpression. Previously published institutional cases were included for complete analysis.^13,14^ The study cohort was subjected to comprehensive medical history, imaging, and histopathologic review to understand the association of specific CNS tumor types, therapeutic responses, and patient outcomes. Detailed genomic, transcriptomic, and epigenomic analyses were performed to elucidate the molecular mechanisms of *FOXR2* activation in specific CNS tumor types and the downstream effects of *FOXR2* activation.

## Materials and Methods

### Study Cohort

Institutional RNA-seq datasets comprising over 3,500 tumors were screened for CNS tumors with *FOXR2* overexpression following batch correction using ComBat and normalization using variance stabilizing transformation. Tumors (*n* = 40) exhibiting *FOXR2* normalized HTSeq counts exceeding the upper quartile (Q_3_) plus 1.5 times the interquartile range (IQR) were selected for review. Samples (*n* = 5) with suboptimal sequencing coverage, *FOXR2* expression levels resembling background noise, or lacking distinct *FOXR2* exonic expression patterns or overexpression in replicates were excluded. The cohort was supplemented with published institutional cases of CNS NB-FOXR2 (*n* = 5) and PB-FOXR2 (*n* = 2).^13,14^ A cohort (*n* = 29) of H3 K27M-mutant diffuse midline glioma (DMG) without *FOXR2* overexpression was assembled for comparison by screening institutional RNA-seq datasets. Clinical data were available for 26 of the 29 cases.

### Transcriptome Sequencing (RNA-seq) and Analysis

RNA was extracted from formalin-fixed paraffin-embedded (FFPE) tissue using a PureLink FFPE RNA Isolation Kit (Thermo Fisher Scientific), as previously described.^15–20^ Purified RNA was quantitated on a Qubit 3 Fluorometer and subjected to RNA-sequencing following the Illumina TruSeq Stranded Total RNA protocol (500 ng RNA minimum). All sequencing data were generated after 100 cycles of paired-end run on an Illumina NovaSeq X Plus. The RNA-seq data were aligned to the human reference genome (hg19).

*FOXR2* structural variants (SVs) were detected by CICERO^21^ or by manually examining sample bam files for soft-clipped reads upstream of the RefSeq *FOXR2* transcript (NM_198451.4). Soft-clipped sequences were used to identify the partner genes involved in the structural alterations by BLAT.^22^ Expressed *FOXR2* exons were identified by manually examining the bam file for peaks in coverage and spliced reads. The exon annotation utilized follows that described in Tsai et al.^2^

RNA feature counts for brain tumor samples from 325 patients were obtained from St. Jude Cloud datasets.^15,23–25^ Normalized counts were used for gene set enrichment analysis (GSEA) using the GSEA Desktop application (version 4.3.3). The analysis parameters included 1,000 permutations and phenotype labels based on activation or non-activation of *FOXR2*.

### Whole Genome Sequencing and Analysis

Genomic DNA was extracted from snap-frozen tumor tissue using an automated Maxwell RSC Instrument (Promega), as previously described.^15,16,26^ DNA quality was assessed on a 4200 TapeStation (Agilent). Paired-end sequencing was conducted on the Illumina HiSeq platform with a 100- or 125-bp read length or NovaSeq with a 150-bp read length. Sequencing results were analyzed using an institutionally established pipeline for alignment and calling of single nucleotide variants and insertions or deletions, which were annotated and ranked by putative pathogenicity using “medal ceremony” and subsequently manually reviewed, as described previously.^27^ DNA breakpoints for *FOXR2* SVs were identified in WGS data (if available) or through intronic RNA soft clips.

### Whole Exome Sequencing and Analysis

Genomic DNA was extracted from FFPE tissue using a QIAamp DNA FFPE Tissue Kit (Qiagen), as previously described.^15,16,18,26^ The genomic libraries were generated using the SureSelectXT kit (Agilent Technologies), followed by exome enrichment using the SureSelectXT Human All Exon V8 bait set (Agilent Technologies). The resulting exon-enriched libraries were subjected to the paired-end, 100-cycle sequencing performed on a NovaSeq X Plus (Illumina). Sequencing results were analyzed as described above.

### Genome-wide DNA methylation profiling and analysis

Genomic DNA was extracted as stated above and subjected to genome-wide methylation profiling on an Illumina Infinium MethylationEPIC platform, as previously described.^15–20^ Differential methylation analysis was performed in R 4.4.0 using the minfi 1.50.0,^28^ limma 3.60.2,^29^ ChAMP 2.29.1,^30^ and sesame 1.22.1^31^ packages with default parameters. The DMRs identified were then used for GSEA by querying against the MSigDB (https://www.gsea-msigdb.org/gsea/msigdb/). Tumor methylation profiles were classified using the Molecular Neuropathology brain tumor classifier (www.molecularneuropathology.org) v12.8. A matching score higher than 0.9 was used as the threshold for classifying molecular tumor type.

### Histopathology Review and Immunohistochemistry

Hematoxylin and eosin-stained 5 μm sections of FFPE tissue specimens of tumor samples were centrally reviewed by a board-certified neuropathologist specialized in pediatric CNS tumors (JC) to confirm the diagnosis. The following antibodies were used for immunohistochemistry: GFAP (DAKO, M0761, clone 6F2, 1:400), Olig2 (Cell Marque, 387M-15, clone 211F1.1, 1:50), and synaptophysin (Leica Microsystems, NCL-L-SYNAO-299, clone 27G12, 1:400).

### Imaging Review

A retrospective review of all baseline and follow-up magnetic resonance imaging (MRI) was performed by a pediatric neuroradiologist (SNP) blinded to the final diagnosis. Baseline tumor location, margins, cellularity, enhancement, and leptomeningeal metastases were recorded. On follow-up, tumors were assessed for pseudoprogression, response to therapy, or progressive disease. Pseudoprogression was defined as the transient increase in tumor size within the first three months after completion of radiation or immunotherapy, which resolved on follow-up imaging.^32,33^ Response to therapy was graded as complete, partial, or minor following the Response Assessment in Pediatric Neuro-Oncology (RAPNO) criteria or, in the case of immunotherapy, the Immunotherapy Response Assessment in Neuro-oncology (iRano) criteria. Progressive disease was defined as a greater than 25% increase in the product of the biperpendicular dimensions of the primary tumor or the presence of new leptomeningeal metastases, which persisted/worsened on follow-up imaging.^34,35^

### Statistical Analysis

Clinical data, including demographic information, initial metastatic staging, and treatment details, was extracted from medical records. The surgery date to obtain diagnostic tissue samples was designated as the date of diagnosis. The date of progression was determined by imaging. Progression was defined by imaging as described above or the conversion of CSF cytology to positive in two consecutive evaluations. Event-free survival (EFS) was defined as the interval from the date of primary tumor diagnosis to the earliest date of disease progression, second malignancy, or death due to any cause for patients who experienced events or to the date of last follow-up for patients without events. Overall survival (OS) was defined as the time interval from the date of diagnosis to the date of death from any cause or to the date of the last follow-up for survivors. Survival outcomes were estimated with the survival package (version 3.3.1) in R 4.2.1 using the Kaplan–Meier method with 95% confidence intervals. Differences in survival distributions were examined by log-rank test.

## Results

### The Landscape of CNS Tumors with *FOXR2* Overexpression

**Figure 1.**
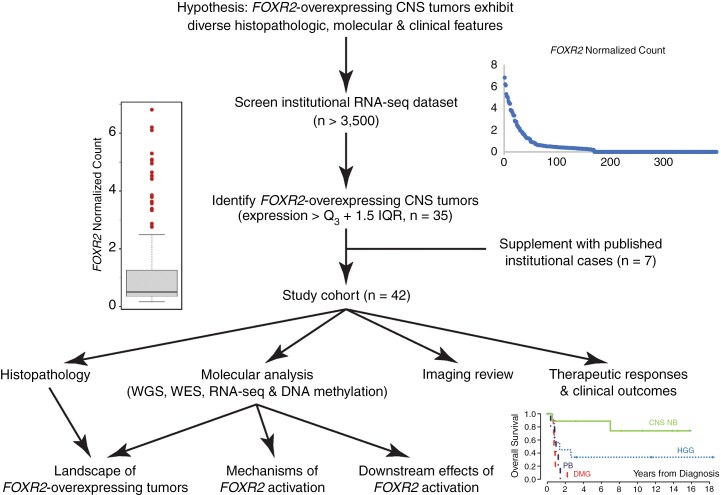
Study design. The Institutional RNA-sequencing (RNA-seq) dataset was screened for tumors that showed significantly elevated, more than 1.5 interquartile range (IQR), *FOXR2* expression. Tumors that showed no apparent exonic structure of the *FOXR2* transcript, likely indicating processing artifacts, were excluded. The 35 tumors identified, supplemented with seven previously published institutional cases, were subjected to comprehensive histologic, molecular, imaging, and clinical data review.

**Figure 2.**
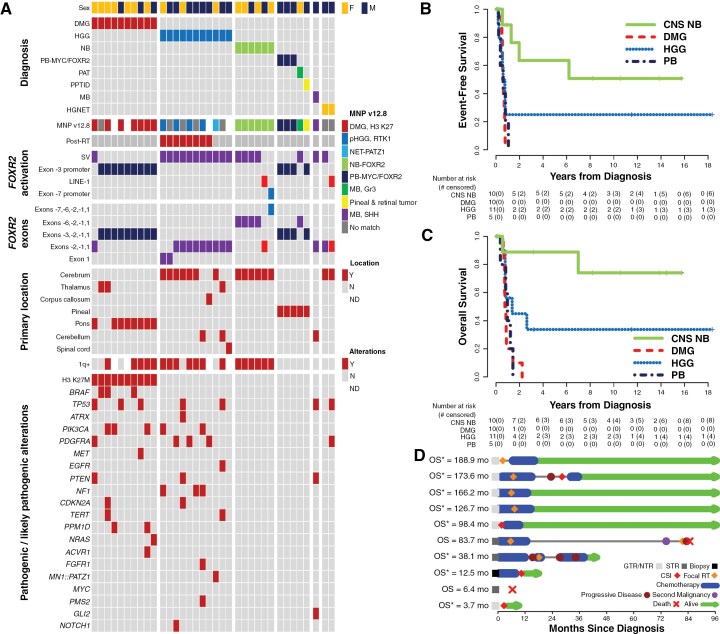
Study cohort characteristics and patient outcomes. (A) Oncoplot summarizing the study cohort’s demographic, histologic, and molecular characteristics. Event-free (B) and overall survival (C) of the four major tumor types in the study cohort. (D) Swimmer plot showing the detailed treatment response and patient outcomes of CNS NB in our cohort. CNS NB: CNS neuroblastoma with *FOXR2* activation. DMG: histone H3 K27M-mutant diffuse midline glioma. HGG: high-grade glioma. PB: Pineoblastoma.

A total of 42 CNS tumors with *FOXR2* overexpression from 41 patients were included in this study, including one patient with CNS NB and a secondary radiation-associated tumor (Figs. 1 and 2, Supplementary Table S1). Among them, 21 (50.0%) were high-grade gliomas (HGGs), 18 (42.9%) were embryonal tumors, two were difficult-to-classify high-grade neuroepithelial tumors (HGNET), and one was pineal parenchymal tumor of intermediate differentiation (PPTID). This finding confirms our hypothesis that CNS tumors with *FOXR2* overexpression comprise tumors of diverse histology. While rare, a PPTID in this cohort suggests that CNS tumors with *FOXR2* overexpression are not necessarily histologically high-grade. There were no significant differences in *FOXR2* expression levels among tumors in different categories (CNS NB-FOXR2 vs. all others, *P* = 0.82).

The HGGs with *FOXR2* overexpression included ten H3 K27M-mutant DMGs, eight radiation-associated secondary HGGs, one with *MN1::PATZ1* fusion, and two not-elsewhere-classified HGGs (Fig. 2A). HGGs with *FOXR2* overexpression harbored diverse concomitant genetic alterations frequently encountered in pediatric HGGs, including DMGs. The two thalamic DMGs harbored concomitant histone H3 K27M and *BRAF* V600E mutations, an infrequent combination in DMGs. One pontine DMG harbored a *KIAA1549::BRAF* fusion, a molecular alteration associated with pilocytic astrocytoma and diffuse leptomeningeal glioneuronal tumor and exceedingly rare in DMGs. Chromosome arm 1q gain (1q+), a characteristic molecular alteration of CNS NB-FOXR2, was seen in 70.6% (12/17) of HGGs with *FOXR2* overexpression tested, demonstrating that the combination of 1q+ and *FOXR2* overexpression is not specific to CNS NB-FOXR2.

The embryonal tumors with *FOXR2* overexpression included 11 CNS NB-FOXR2, six pineoblastoma (PB), including five PB-FOXR2 and one pineal anlage tumor (PAT), and one medulloblastoma (MB). Therefore, *FOXR2* overexpression is not limited to PB-FOXR2 in the pineal region. Except for the one SHH-activated and *TP53*-mutant MB, all other embryonal tumors with *FOXR2* overexpression did not harbor other pathogenic or likely pathogenic genetic alterations.

All CNS NB-FOXR2 were cerebral cortical tumors, while other CNS tumors with *FOXR2* overexpression show regional predilections depending on the specific tumor type. Our results support our hypothesis that CNS tumors with *FOXR2* overexpression exhibit diverse histopathologic, molecular, and clinical features.

### Patient Outcomes

Forty patients had detailed clinical information for analysis. Demographic information, histological diagnosis, the extent of resection, metastatic staging at diagnosis, and treatment regimens are summarized in SupplementaryTable S2 and SupplementaryFig. S1. The median age at diagnosis of the entire cohort was 4.5 years (0.50–22.28 years) and varied with underlying diagnosis. Eighteen (44%) were females. Age, metastatic status at diagnosis, and tumor location were associated with histological diagnosis (SupplementaryTable S2). Treatment varied significantly based on the age of diagnosis and histological diagnosis, with the majority receiving multimodal therapy upfront. Event-free (EFS) and overall survival (OS) based on diagnosis are depicted in Fig. 2B and C.

#### CNS NB-FOXR2

Ten patients had CNS NB-FOXR2 with clinical data available. The median age at diagnosis was 5.3 (range: 1.6–13.6 years). All tumors were supratentorial/hemispheric and non-metastatic at diagnosis. Two patients remained in upfront therapy at the time of analysis. Overall outcomes were significantly better than all other tumor types with *FOXR2* overexpression. Treatment with multimodal therapy appears to be crucial, as one patient with no therapy had rapid progression and died. One other patient died due to a secondary HGG with no recurrence of CNS NB. (Fig. 2D)

#### DMG

Ten patients had DMG with *FOXR2* overexpression. Their demographics and clinical parameters were compared with an institutional cohort of 26 DMGs without *FOXR2* activation (Suppl Table 3). Patients with DMG with *FOXR2* overexpression were younger at diagnosis (6.0 vs 9.1 years, *P* = 0.007), but rates of metastatic disease at diagnosis, gender, and extent of resection were similar. DMGs with *FOXR2* overexpression included eight pontine and two thalamic tumors, with 1-year EFS at 0% and OS at 20%. The DMGs without *FOXR2* activation included 18 pontine, five thalamic, one spinal, and two other locations, with 1-year EFS and OS at 3.9% and 46.2%, with no significant difference between those with and without *FOXR2* activation (SupplementaryFig. S2). All patients received radiation, and adjuvant or targeted therapies showed no survival benefit.

#### HGG

Eleven patients had non-DMG HGG, including eight radiation-induced and three primary tumors. All received radiation and predominantly temozolomide-based chemotherapy. Two patients with radiation-induced HGGs and one with *MN1::PATZ1* fusion are long-term survivors. Two are recently diagnosed and undergoing treatment. The remaining patients experienced rapid disease progression and poor outcomes despite treatment.

#### PB

Six patients with PB with *FOXR2* overexpression (five classic PB and one pineal anlage tumor) had a mean age of diagnosis of 1.7 years (0.5–5.7 years). All received upfront multimodal chemotherapy, with radiation administered to older patients. Survival outcomes were poor, with 0% OS at two years and frequent progression during upfront therapy.

#### Other

Our cohort included two HGNETs, one MB, and one PPTID. Both HGNET patients were under two years old, non-metastatic at diagnosis, and treated with upfront chemotherapy alone; one experienced disease progression and died, while the other remained in upfront therapy. The medulloblastoma and PPTID patients received radiation and chemotherapy and are alive without evidence of disease.

### Histologic spectrum of CNS NB-*FOXR2*

**Figure 3.**
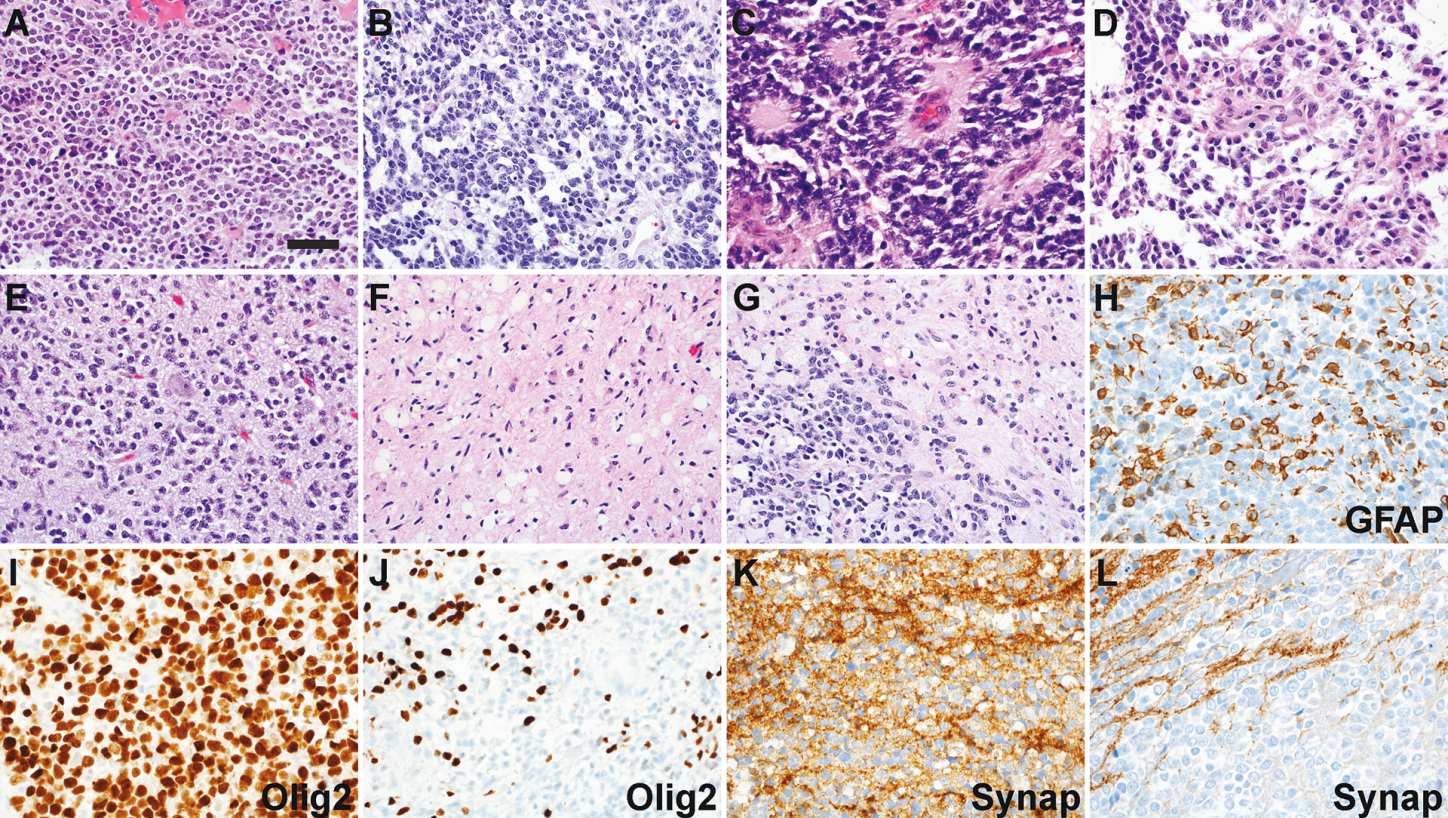
Histologic spectrum of CNS NB-FOXR2. (A–C) The characteristic embryonal-looking areas of CNS NB-FOXR2 showed substantial variation. Areas resembling high-grade glioma may be found (D). Diffusely infiltrating tumor cells may masquerade as a glial neoplasm (E). Some tumors contained low-grade appearing regions (F), but there were nodules of embryonal cells (G). There may be genuine GFAP expression among the tumor cells (H). The typical diffuse Olig2 (I) and synaptophysin (Synap, K) expression could be patchy (J, L) instead. Scale bar: 100 microns

While DMGs, HGGs, PBs, MB, and PPTID with *FOXR2* overexpression in our cohort exhibited typical histopathologic features of their specific tumor type, *FOXR2*-activated CNS NB demonstrated substantial variability in histology and immunophenotype (Fig. 3). All tumors contained areas exhibiting embryonal cytology, with poorly differentiated cells with abundant mitotic activity, high nuclear-to-cytoplasmic ratios, hyperchromatic nuclei, and inconspicuous amounts of cytoplasm. None showed ganglion cell differentiation. The architecture of the embryonal areas varied (Fig. 3A–C). Two samples of small biopsy displayed only embryonal tumor appearance, and the rest with large resections contained regions showing variable glial characteristics, including more prominent eosinophilic cytoplasm (Fig. 3D), infiltrative growth (Fig. 3E), fibrillary background (Fig. 3F), and mixed high-grade and low-grade appearance (Fig. 3G). Immunohistochemistry detected various extents of genuine glial fibrillary acidic protein (GFAP) expression by the tumor cells (Fig. 3H). While typical CNS NB demonstrates extensive oligodendrocyte transcription factor 2 (Olig2) and synaptophysin expression, there was substantial variability (Fig. 3I–L).

### Imaging Features of CNS Tumors with *FOXR2* Overexpression

A blinded review was performed for 35 tumors from 34 patients with baseline imaging available, including one patient who initially presented with CNS NB and then developed a secondary post-RT HGG. A total of 21 tumors had imaging features suggestive of HGGs, seven were suggestive of CNS NB, six were suggestive of PB, and one was suggestive of either PB or PPTID. Key baseline imaging features are summarized in Supplementary Table S4. Imaging diagnosis was based on typical characteristics of CNS NB, HGG, PB, or PPTID without any relevance to their *FOXR2* activation status. There was 100% concordance between the suspected diagnosis from the blinded imaging review and the final diagnosis.

**Figure 4.**
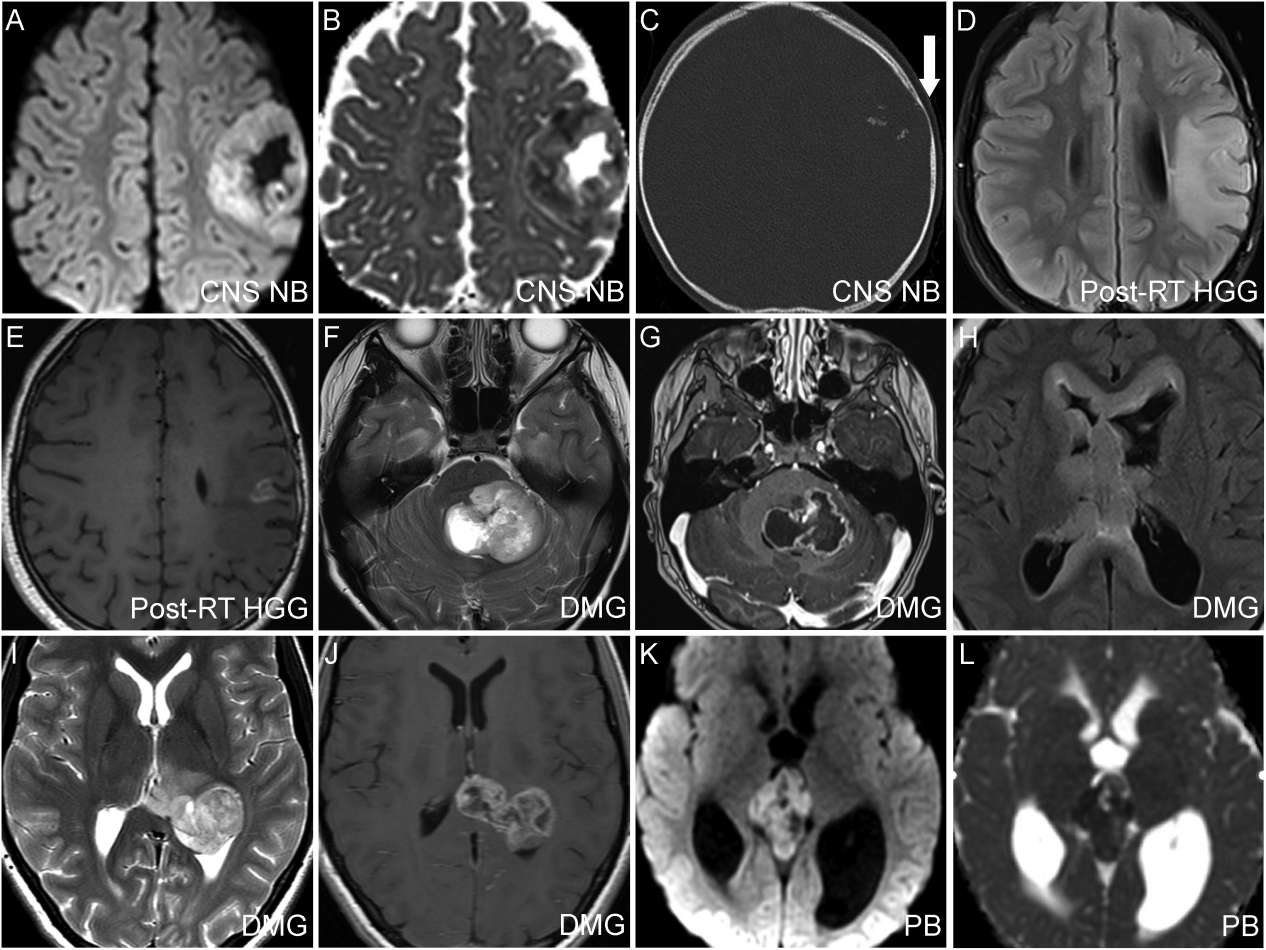
Imaging features of CNS tumors with *FOXR2* overexpression. Baseline axial diffusion-weighted (A), Apparent Diffusion Coefficient (ADC) map (B), and CT (C) images showing a hypercellular left frontal CNS NB-FOXR2 with coarse calcifications and cortical remodeling, suggestive of a CNS NB-FOXR2 (C, arrow). Axial FLAIR (D) and post-contrast T1 (E) images showing an infiltrative left frontal high-grade glioma (HGG) with enhancement several years after radiation therapy for a CNS NB-FOXR2 at the same location. Axial T2 (F), and post-contrast T1 (G) images showing a heterogeneously enhancing left pontine diffuse midline glioma (DMG). (H) Four months post-RT fluid-attenuated inversion recovery (FLAIR) image showing extensive new subependymal metastases. (I) Axial T2 and post-contrast T1 (J) images showing a circumscribed and heterogeneously enhancing left dorsal thalamic DMG. (K) Baseline diffusion-weighted images and (L) ADC map showing a hypercellular pineal mass, suggestive of a pineoblastoma.

The seven CNS NBs were all in the frontal lobe and universally well-circumscribed, showed diffusion restriction, and were cortically based with minimal white matter extension. (Fig. 4). In contrast, the seven hemispheric HGGs exhibited infiltrative margins, extensive white matter involvement, and variable hypercellularity and enhancement.

On follow-up imaging, pseudoprogression was noted in four pontine DMGs, either following radiation (three) or immunotherapy (one), and was absent in other tumors. These four cases of pontine DMGs eventually exhibited progressive disease approximately one to four months after pseudoprogression resolved.

### Mechanisms of *FOXR2* Activation

**Figure 5.**
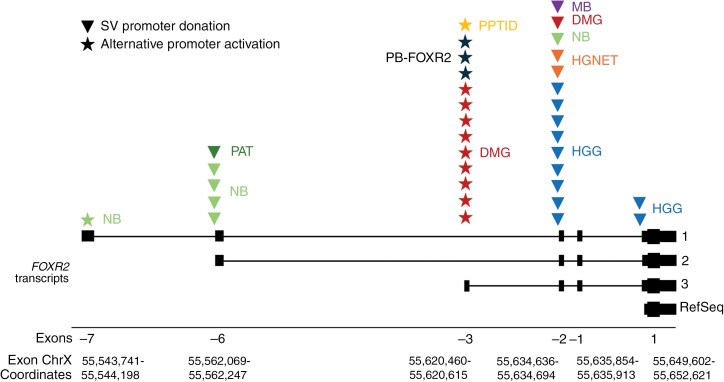
Mechanisms of *FOXR2* activation. Summary of structural variant (SV) promoter donation and *FOXR2* promoter activation events observed per tumor type. SV events occur as gene fusions to exons-6, -2, or 1, while promoter activation events occur at exon-3 or -7. *FOXR2* transcripts 1–3 are based on the splicing pattern seen in our cohort of activated tumors, and the exons are annotated as in Tsai et al.^2^ The RefSeq *FOXR2* transcript has only one exon that starts > 200 bp downstream of the exon 1 splice site in activated transcripts 1–3. The coding region of *FOXR2* occurs within exon 1 and is identical for all transcripts. Exon 1 coordinates are for transcripts 1–3, not RefSeq.

In our cohort, the most common mechanisms of *FOXR2* activation involved structural alterations causing promoter donation from other genes and incorporating upstream non-canonical, non-coding exons of *FOXR2*, followed by alternative promoter activation or truncated LINE-1 retrotransposition (Fig. 5, Supplementary Figs. 3–7). Structural alterations resulting in promoter donation occurred as fusions to *FOXR2* exon-6 (*n* = 5), -2 (*n* = 14), or 1 (*n* = 2). Alternative promoter activation events primarily occurred at the exon-3 promoter (*n* = 13), resulting in the expression of *FOXR2* transcript 3. Only one case had apparent activation of the exon-7 promoter, resulting in the expression of *FOXR2* transcript 1. Two exon-2 alterations resulted from recurrent truncated LINE-1 retrotransposon insertions.

The preferential mechanisms of *FOXR2* activation varied by tumor type (Fig. 5). DMGs (*n* = 9) and pineal tumors (*n* = 4) preferred activation of the exon-3 promoter, with only one DMG and one pineal tumor having a structural event (exon-2 and -6, respectively). The pineal tumor with structural alteration was of a different type (pineal anlage tumor) than the others (PB-FOXR2, PPTID), which could explain the mechanistic difference. Structural alterations were exclusively observed for non-DMG HGGs (exon-2, *n* = 9; exon 1, *n* = 2), HGNETs (exon-2, *n* = 2), and MB (exon-2, *n* = 1). CNS NBs also prefer structural events (exon-6, *n* = 4; exon-2, *n* = 1), with one exceptional case having exon-7 promoter activation.

One of the HGNET cases in our cohort was previously described, with a LINE-1 insertion event that resulted in promoter donation and *FOXR2* activation (SJHGG030242).^9^ We found another case in our cohort (SJBT032161_D1) with the same RNA soft clips at *FOXR2* exon-2, supporting a LINE-1 insertion event (Supplementary Fig. S7). This was the only CNS NB case with a structural alteration resulting in promoter donation to *FOXR2* exon-2, as the rest preferred *FOXR2* exon-6.

Analysis of RNA coverage and spliced *FOXR2* reads suggested that there are at least two transcripts not currently available in RefSeq that are utilized in the activation of *FOXR2* (transcripts 1 and 3, Fig. 5; Supplementary Fig. S3). All exons upstream of exon 1 appear to be non-coding, as there was no larger open reading frame present in the transcript sequences. All non-coding exon boundaries for these transcripts had the canonical GT/AG sequence at their splice sites, aside from the exon-7/exon-6 boundary, which had a GC/AG. This non-canonical junction sequence has previously been observed in humans at a low frequency.^36^  *FOXR2* exon 1 in the activated transcripts has a splice acceptor > 200 bp upstream of the RefSeq transcript (Fig. 5). All *FOXR2*-activated tumors in our cohort utilize this splice site.

The overexpression of *FOXR2*, either through structural variation or alternative promoter activation, was associated with increased CpG methylation levels at the five CpG sites covered by the Infinium MethylationEPIC v2.0 BeadChip at the “gene body” region of the aberrant, non-canonical *FOXR2* transcripts (Supplementary Fig. S8), which has been associated with elevated gene expression throughout the human genome.^37,38^ Among the five CpG sites at the *FOXR2* locus, three (cg09113017, cg17779979, and cg26217484) showed significantly increased methylation levels in *FOXR2*-activated tumors, while two (cg01704778 and cg25478472) retained their high basal methylation levels. Other profiled CpG sites at nearby genes (*USP51* and *RRAGB*) showed no changes in methylation levels, suggesting that changes at the *FOXR2* locus are specific to *FOXR2* activation.

### Expressions Patterns of Genes That Donated Their Promoters

Expression analysis using GTEx data (accessed Aug 1, 2024) showed that most partner genes identified in our study, except for *FAM156A*, had low-moderate to highly elevated expression in different tissues and were overall enriched in the brain (Supplementary Fig. S9). We also analyzed H3K27ac and H3K4me3 marks across normal brain samples from Encode (ENCFF190JDV; ENCFF717CEO), which showed the SV breakpoints in the partner genes to be associated with enhancers or promoters in almost all cases, as defined by a peak within 25 kb (Supplemental Table 5).

*FOXR2* partner genes that donate their promoters or enhancer elements perform a diverse array of functions (Suppl Table 6), including in RNA processing and regulation (*RBM10*, *ELAVL2*), transcriptional regulation and epigenetic modification (*BCOR*, *FTX*, *KMT5B*, *MED13L*, *FAM156A*), the ubiquitin-proteosome system (*MARCHF6*, *RNF144A*, *USP51*, *TBL1XR1*), neural development and synaptic function (*CASK*, *NLGN4X*, *ZC4H2*), and cell signaling transduction (*TAB2*, *ITFG1*, *CHM*). Most partner genes have roles in various stages of brain development and, when mutated in the germline, cause a spectrum of syndromic and non-syndromic neurodevelopmental disorders.

### Downstream Effects of *FOXR2* Activation in CNS Tumors

#### Epigenetic effect of FOXR2 activation in CNS tumors

We first looked at the downstream effects of *FOXR2* activation in CNS tumors at the DNA methylation level by comparing the DNA methylome profiles of tumors with *FOXR2* overexpression in our cohort (*n* = 30 with sufficient data quality) with those of DMGs without *FOXR2* activation (*n* = 25). Differential methylation analysis identified 10,002 differentially methylated regions (DMRs) between these cohorts. GSEA of genes in the DMRs identified enrichment of FOXR2 target genes and downstream targets of several transcription factors (Suppl Table 7). Notably, the analysis results suggested the activation of MYC and the ETS family transcription factors ETS1, ELF2, ELF5, ELK1, GABPA, GABPB2, NRF2, CETS1P54, DIDO1, and EPC1 in CNS tumors with *FOXR2* overexpression, as suggested by previous studies in various tumor types.^2,39,40^

### Transcriptome Analysis Further Supports the Activation of MYC and ETS Pathways

**Figure 6.**
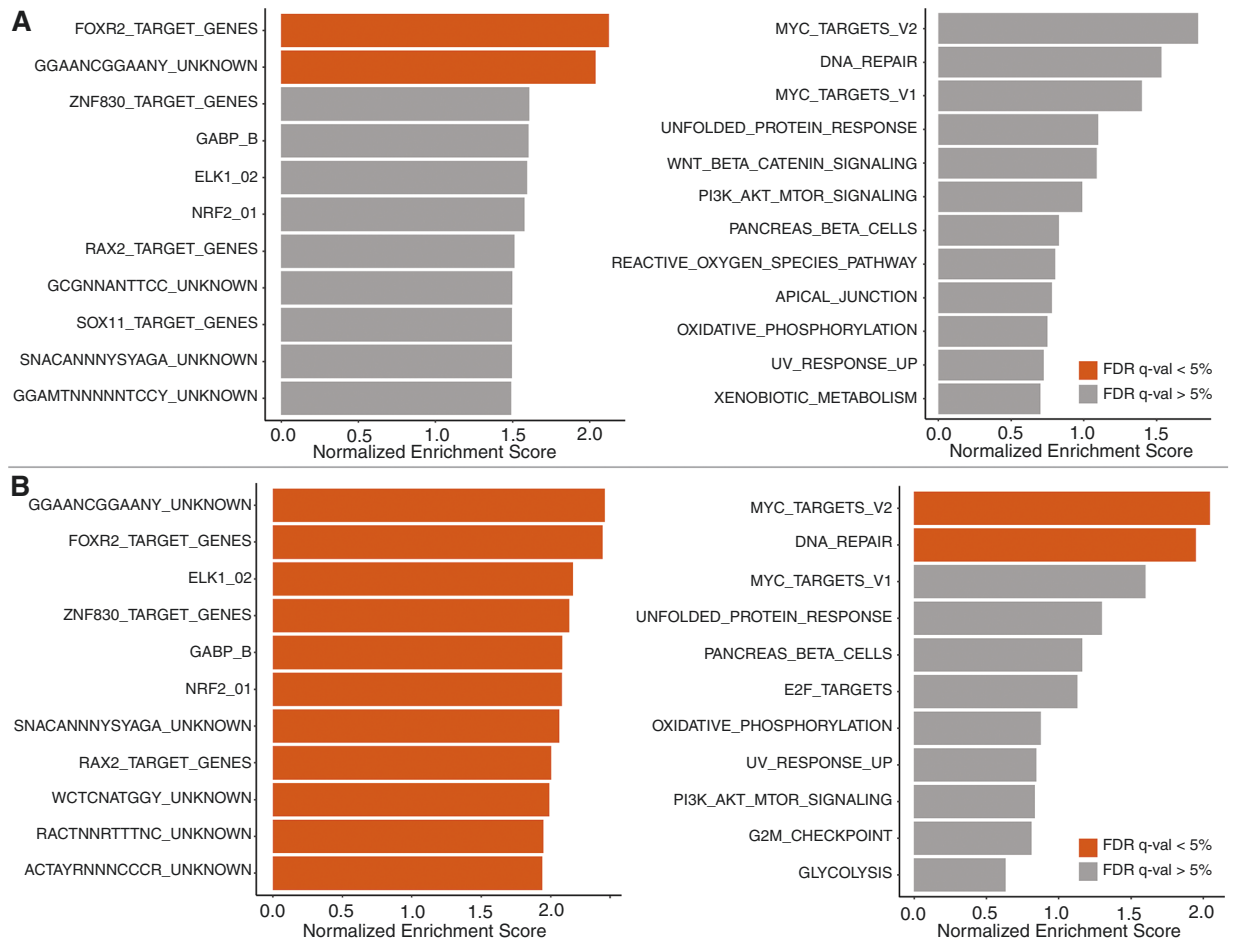
Gene set enrichment analysis. (A) Results of *FOXR2*-activated histone H3 K27M-mutant diffuse midline glioma (*n* = 10) vs non-activated tumors (*n* = 29). (Left) Results with regulatory target gene sets with a total of 337/3160 gene sets showing upregulation in the activated group and a total of 2 gene sets are significant at FDR < 5%. (Right) Results with hallmark gene set show that a total of 35/50 gene sets are upregulated in the activated group, and no gene sets are significant at FDR < 5%. (B) Results of *FOXR2*-activated CNS tumors (*n* = 32) vs non-activated tumors (*n* = 303). (Left) Results with regulatory target gene sets with a total of 401/3160 gene sets show upregulation in the activated group, and a total of 18 gene sets are significant at FDR < 5%. (Right) Results with hallmark gene set show that a total of 11/50 gene sets are upregulated in the activated group, and two gene sets are significant at FDR < 5%.

We then compared the transcriptome profiles of DMGs with *FOXR2* overexpression (*n* = 10) with non-*FOXR2*-activated DMGs (*n* = 29), as well as the profiles of all tumors with *FOXR2* overexpression (*n* = 32) with brain tumors lacking *FOXR2* activation (*n* = 303). While the DMG cohorts were smaller, GESA of differentially expressed genes of both comparisons identified the enrichment of FOXR2 target genes (Fig. 6) and suggested the activation of the MYC pathway and the ETS family transcription factors (ELK1, GABPA, GABPB2, and NRF2). This result is consistent with the methylome findings and suggests the activation of MYC and ETS family transcription factors is a common feature of CNS tumors with *FOXR2* overexpression.

## Discussion

Our findings support the hypothesis that CNS tumors with *FOXR2* overexpression exhibit diverse histopathologic, molecular, and clinical features, indicating that *FOXR2* alterations are not pathognomonic of CNS NB-FOXR2. Instead, a broad spectrum of HGGs, including DMGs, CNS embryonal tumors (CNS NB, PB, PAT, and MB), and PPTID can harbor *FOXR2* activation. Our study also reveals a unique enrichment of radiation-induced HGGs with *FOXR2* activation, a previously unreported finding.

Clinical characteristics and outcomes differed across the various tumor types. CNS NB occurred in patients with a median age of ~6 years and overall had excellent outcomes. The favorable overall survival aligns with other published series, including a European report where PFS and OS in a cohort of 63 patients were 63% and 85%, respectively.^41^ Like our cohort, treatment varied, and RT appears crucial for good outcomes. However, the optimal RT dose remains unclear, given the excellent survival with therapy. Given the low incidence of leptomeningeal dissemination, the possibility of a lower CSI dose in older patients and focal RT in younger patients should be explored in future clinical trials.

The findings of our blinded imaging review were consistent with the reported imaging characteristics of *FOXR2-*activated CNS NB.^42^ The distinctive imaging features can help differentiate CNS NB from other embryonal tumors, such as embryonal tumors with multilayered rosettes, which have prominent intra-tumoral veins and hypo-enhancement.^43^ On follow-up imaging, pseudoprogression was only observed in a subset of pontine DMGs and was notably absent in thalamic, spinal, and hemispheric HGGs. This unique behavior of pontine DMG with *FOXR2* overexpression in our cohort may suggest an underlying susceptibility to radiation- and immunotherapy-mediated inflammation and needs further exploration in prospective studies.

Our findings reveal three mechanisms for aberrant *FOXR2* activation in CNS tumors—promoter donation/enhancer hijacking through structural rearrangement, alternative promoter activation, and truncated LINE-1 retrotransposition, which showed differential preference among the various tumor types and anatomical sites. Structural rearrangement is the preferred mechanism of *FOXR2* activation in cerebral tumors, including non-DMG HGGs and CNS NB. In contrast, midline (thalamic, pontine, and pineal) tumors, including DMG, PB, and PPTID, prefer utilizing alternative promoters. HGGs and DMGs with *FOXR2* overexpression additionally harbor concomitant genetic alterations frequently encountered in HGGs. These findings may have diagnostic utility in cases with ambiguous histology. For example, one of the HGNETs in our cohort, while challenging to classify based on histology, harbored concomitant *TP53* alteration and *PDGFRA* amplification, findings associated with HGGs rather than CNS NB. One RT-associated HGG with *FTX::FOXR2* fusion occurred after RT for a primary CNS NB with *BCOR::FOXR2* fusion, harbored *NF1* and *PIK3CA* alterations, findings typically associated with HGGs. Both tumors progressed rapidly despite therapy, an outcome distinct from typical CNS NB.

Based on the NCBI Reference Sequence Database (RefSeq), *FOXR2* is a single-exon gene in humans. However, we observed the inclusion of upstream non-canonical exons in the *FOXR2* transcripts in CNS tumors with *FOXR2* overexpression. A splice acceptor upstream of exon 1 is utilized in all tumors with *FOXR2* overexpression. These findings must be considered when looking for aberrant *FOXR2* activation by RNA-sequencing or similar techniques in CNS tumors.

No new protein variant is predicted to be generated from the aberrant *FOXR2* fusion transcripts in all tumors with *FOXR2* overexpression in our cohort, which drastically differs from conventional gene fusions, where a hybrid protein with functional domains from each partner is brought together, resulting in new proteins with gain of function. This finding suggests that direct interaction with MYC through its MYC-binding domain, located near the N-terminus of FOXR2, and specific DNA binding through its Forkhead domains near its C-terminus are required for FOXR2-mediated tumorigenesis. This observation and the finding that both MYC pathway and ETS family transcription factors are commonly activated in CNS tumors with *FOXR2* overexpression may offer a unique therapeutic opportunity. Concomitant alterations in DMGs and HGGs with *FOXR2* overexpression, including *BRAF*, *PDGFRA*, *EGFR*, *FGFR1*, *PPM1D*, *NRAS*, *PIK3CA*, *PTEN*, and *ACVR1*, offer additional therapeutic opportunities.

Our study identified a second example of aberrant *FOXR2* activation driven by a truncated LINE-1 retrotransposition event,^9^ suggesting that such an oncogenic mechanism is a recurrent finding. The LINE-1 target site (5’-GTTGATATCTTT) located upstream of exon-2 was likely utilized in both tumors since the resulting aberrant *FOXR2* transcripts share a similar structure. In both tumors, the LINE-1 promoter drives the transcription of a chimeric *LINE-1::FOXR2* transcript as the only driver event present at diagnosis. Our finding again supports LINE-1 promoter donation as a novel cancer-initiating driver mechanism, as opposed to previously reported LINE-1-mediated disruption of tumor suppressors or oncogene repressors.^44^

In summary, our findings demonstrate that CNS tumors with *FOXR2* overexpression exhibit diverse histopathologic, molecular, and clinical features. Specific tumor types are associated with preferential mechanisms of *FOXR2* activation, utilization of upstream non-canonical non-coding exons, and patient outcomes. There is an enrichment of RT-associated secondary tumors and concomitant genetic alterations in HGGs and DMGs with *FOXR2* overexpression. CNS NB-FOXR2 demonstrates favorable outcomes, and therapy de-escalation should be further investigated in future clinical trials.

## Supplementary material

Supplementary material is available online at *Neuro-Oncology* (https://academic.oup.com/neuro-oncology).

noaf076_suppl_Supplementary_Tables_1-7_Figures_1-9

## Data Availability

Once the manuscript is published, the raw and processed data will be available at the St. Jude Cloud23 (https://www.stjude.cloud/).
