# Supplementary material for: FOXR2 activation is not exclusive of CNS neuroblastoma
Source: Neuro Oncol. 2025 Apr 15;27(7):1801–12. doi: 10.1093/neuonc/noaf076 (PMC12417820; doi:10.1093/neuonc/noaf076)
Supplement: noaf076_suppl_Supplementary_Tables_1-7_Figures_1-9 [file noaf076_suppl_supplementary_tables_1-7_figures_1-9.zip › Suppl Table 2_Baseline Characteristics_20250107.docx]

**Supplementary Table 2 Baseline characteristics**

|  | **CNS NB (10)** | **DMG (10)** | **RT-HGG (8)** | **HGG, Other (3)** | **PB (6)** | **PPTID (1)** | **MB (1)** | **HGNET (2)** |
| --- | --- | --- | --- | --- | --- | --- | --- | --- |
| **Age, mean**  **(range)** | 5.3  (1.6-13.6) | 6.0  (3.3-16.4) | 15.2  (7.8-22.3) | 11.6  (0.9-17.8) | 1.7  (0.5-5.7) | 12.0 | 8.6 | 1.1  (0.9-1.21) |
| **Gender** |  | | | | | | | |
| Male | 4 (40%) | 3 (30%) | 6 (75%) | 2 (67%) | 4 (67%) | 1 (100%) | 1 (100%) | 2 (100%) |
| Females | 6 (60%) | 7 (70%) | 2 (25%) | 1 (33%) | 2 (33%) |  |  |  |
| **Location** |  | | | | | | | |
| Hemispheric | 10 | 0 | 6 | 1 | 0 | 0 | 0 | 2 |
| Thalamic/Mid | 0 | 2 | 1 | 1 | 0 | 0 | 0 | 0 |
| Pineal | 0 | 0 | 0 | 0 | 6 | 1 | 0 | 0 |
| Brainstem | 0 | 8 | 1 | 0 | 0 | 0 | 0 | 0 |
| Cerebellum | 0 | 0 | 0 | 0 | 0 | 0 | 1 | 0 |
| Spine | 0 | 0 | 0 | 1 | 0 | 0 | 0 | 0 |
| **Extent of Resection** |  | | | | | | | |
| GTR/NTR | 6 | 0 | 2 | 0 | 5 | 1 | 1 | 2 |
| STR | 3 | 2 | 4 | 3 | 0 | 0 | 0 | 0 |
| Biopsy | 1 | 8 | 2 | 0 | 1 | 0 | 0 | 0 |
| **Metastasis Status** |  | | | | | | | |
| M0 | 10 | 9 | 8 | 2 | 3 | 1 | 0 | 2 |
| M+ | 0 | 1 | 0 | 1 | 3 | 0 | 1 | 0 |
| **Up-front Therapy** |  | | | | | | | |
| Radiation  Focal RT  CSI | 5  3 | 9  1 | 7  0 | 1  1 | 2  1 | 1  0 | 0  1 | 0  0 |
| Adjuvant  Chemo with RT | 8 | 5 | 5 | 1 | 3 | 1 | 1 | 0 |
| Chemo only | 1 | 0 | 1 | 1 | 3 | 0 | 0 | 2 |
| None | 1 | 0 | 0 | 0 | 0 | 0 | 0 | 0 |
