# Supplementary material for: FOXR2 activation is not exclusive of CNS neuroblastoma
Source: Neuro Oncol. 2025 Apr 15;27(7):1801–12. doi: 10.1093/neuonc/noaf076 (PMC12417820; doi:10.1093/neuonc/noaf076)
Supplement: noaf076_suppl_Supplementary_Tables_1-7_Figures_1-9 [file noaf076_suppl_supplementary_tables_1-7_figures_1-9.zip › Suppl Table 3_DMG cohort_20241104.docx]

**Supplementary Table 3 DMGs with and without *FOXR2* activation**

|  | **Total (36)** | ***FOXR2*-activated(10)** | **Not *FOXR2*-activated (26)** | **p-value** |
| --- | --- | --- | --- | --- |
| **Age at Diagnosis (years)** |  |  |  | **0.007** |
| Mean (SD) | 8.3 (3.8) | 6.0 (4.2) | 9.1 (3.2) |  |
| Median (Range) | 8.0 (3.3 – 16.4) | 4.2 (3.3 – 16.4) | 9.1 (3.5 – 14.5) |  |
| **Gender** |  |  |  | 0.274 |
| Female | 19 (52.8%) | 7 (70.0%) | 12 (46.2%) |  |
| Male | 17 (47.2%) | 3 (30.0%) | 14 (53.8%) |  |
| **Metastasis** |  |  |  | 1.000 |
| Yes | 5 (13.9%) | 1 (10.0%) | 4 (15.4%) |  |
| No | 31 (86.1%) | 9 (90.0%) | 22 (84.6%) |  |
| **M Stage** |  |  |  | 1.000 |
| M0 | 31 (86.1%) | 9 (90.0%) | 22 (84.6%) |  |
| M2 | 2 (5.6%) | 0 | 2 (7.7%) |  |
| M3 | 3 (8.3%) | 1 (10.0%) | 2 (7.7%) |  |
| **Resection** |  |  |  | 0.683 |
| GTR/NTR | 1 (2.8%) | 0 | 1 (3.8%) |  |
| STR | 4 (11.1%) | 2 (20.0%) | 2 (7.7%) |  |
| Biopsy | 31 (86.1%) | 8 (80.0%) | 23 (88.5%) |  |
| **Upfront CSI** |  |  |  | 1.000 |
| Yes | 4 (11.1%) | 1 (10.0%) | 3 (11.5%) |  |
| No | 32 (88.9%) | 9 (90.0%) | 23 (88.5%) |  |
| **Upfront Focal RT** |  |  |  | 1.000 |
| Yes | 32 (88.9%) | 9 (90.0%) | 23 (88.5%) |  |
| No | 4 (11.1%) | 1 (10.0%) | 3 (11.5%) |  |
