# Supplementary material for: FOXR2 activation is not exclusive of CNS neuroblastoma
Source: Neuro Oncol. 2025 Apr 15;27(7):1801–12. doi: 10.1093/neuonc/noaf076 (PMC12417820; doi:10.1093/neuonc/noaf076)
Supplement: noaf076_suppl_Supplementary_Tables_1-7_Figures_1-9 [file noaf076_suppl_supplementary_tables_1-7_figures_1-9.zip › Suppl Table 4_Key baseline imaging features_20241207.docx]

**Supplementary Table 4 Key baseline imaging features of *FOXR2*-activated CNS tumors**

| **Imaging feature** | **Location** | **Diffusion restriction** | **Margins** | **Degree of enhancement** | **Degree of mass effect** | **Leptomeningeal disease at presentation** |
| --- | --- | --- | --- | --- | --- | --- |
| CNS NB (7) | Frontal lobe (7) | 7 (100%) | Circumscribed- 7 (100%) | Moderate- 4 (56%)  Marked -2 (29%)  Missing- 1 (15%) | Mild -5 (71%)  Severe -2 (29%) | 0 (0%) |
| HGG,  hemispheric (7) | Frontal lobe only (3)  Frontal and parietal lobes (2)  Frontal and temporal lobes (1)  Deep white matter of bilateral parietal lobes (1) | 3 (43%) | Infiltrative- 7 (100%) | Marked- 3 (43%)  Moderate- 2 (29%)  Mild to none- 2 (29%) | Mild to none – 4 (56%)  Moderate – 3 (43%) | 0 (0%) |
| HGG,  midline or paramedian (14) | Thalami (4)  Pons (9)  Cervical spine (1) | 4 (28%) | Infiltrative- 9 (64%)  Circumscribed- 5 (36%) | Moderate- 9 (64%)  Mild to none -5 (36%) | Moderate- 10 (72%)  Severe – 2 (14%)  Mild to none- 2 (14%) | 2 (14%) |
| PB (6) | Pineal region-6 | 6 (100%) | Circumscribed-6 (100%) | Marked – 5 (80%)  Mild- 1 (20%0 | Moderate- 6 (100%) | 3 (50%) |
| PB/PPTID (1) | Pineal region | 1 | Circumscribed | Marked | Moderate | 0 (0%) |
