# Supplementary material for: FOXR2 activation is not exclusive of CNS neuroblastoma
Source: Neuro Oncol. 2025 Apr 15;27(7):1801–12. doi: 10.1093/neuonc/noaf076 (PMC12417820; doi:10.1093/neuonc/noaf076)
Supplement: noaf076_suppl_Supplementary_Tables_1-7_Figures_1-9 [file noaf076_suppl_supplementary_tables_1-7_figures_1-9.zip › Suppl Table 5_H3K27ac_ H3K4me3_20241104.docx]

**Supplementary Tale 5 The presence of H3K27ac and H3K4me3 mark peaks within 25kb of the structural variation breakpoints among the *FOXR2* partner genes in normal brain samples according to Encode, suggesting the association with enhancers or promoters**

| ***FOXR2* Partner Gene** | **Mark** | **Source** | **H3K27Ac Coverage: Brain - Frontal Cortex (BA9)- GTEx** | **Peak within 25 kb** |
| --- | --- | --- | --- | --- |
| *CHM* | H3K4me3 | ENCFF717CEO |  | Yes |
| *FTX* | H3K4me3 | ENCFF717CEO |  | Yes |
| *ZC4H2* | H3K4me3 | ENCFF717CEO |  | Yes |
| *NLGN4X* | H3K4me3 | ENCFF717CEO |  | Yes |
| *BCOR* | H3K4me3 | ENCFF717CEO |  | No |
| *CASK* | H3K4me3 | ENCFF717CEO |  | Yes |
| *RBM10* | H3K4me3 | ENCFF717CEO |  | Yes |
| *FAM156A* | H3K4me3 | ENCFF717CEO |  | Yes |
| *USP51* | H3K4me3 | ENCFF717CEO |  | Yes |
| *RNF144A* | H3K4me3 | ENCFF717CEO |  | Yes |
| *TBL1XR1* | H3K4me3 | ENCFF717CEO |  | Yes |
| *MARCHF6* | H3K4me3 | ENCFF717CEO |  | Yes |
| *TAB2* | H3K4me3 | ENCFF717CEO |  | Yes |
| *ELAVL2* | H3K4me3 | ENCFF717CEO |  | Yes |
| *KMT5B* | H3K4me3 | ENCFF717CEO |  | Yes |
| *MED13L* | H3K4me3 | ENCFF717CEO |  | Yes |
| *ITFG1* | H3K4me3 | ENCFF717CEO |  | Yes |
|  |  |  |  |  |
| *CHM* | H3K27Ac | ENCFF190JDV | Yes | Yes |
| *FTX* | H3K27Ac | ENCFF190JDV | Yes | Yes |
| *ZC4H2* | H3K27Ac | ENCFF190JDV | Yes | No |
| *NLGN4X* | H3K27Ac | ENCFF190JDV | Yes | Yes |
| *BCOR* | H3K27Ac | ENCFF190JDV | Yes | Yes |
| *CASK* | H3K27Ac | ENCFF190JDV | Yes | Yes |
| *RBM10* | H3K27Ac | ENCFF190JDV | Yes | Yes |
| *FAM156A* | H3K27Ac | ENCFF190JDV | Yes | Yes |
| *USP51* | H3K27Ac | ENCFF190JDV | Yes | Yes |
| *RNF144A* | H3K27Ac | ENCFF190JDV | Yes | Yes |
| *TBL1XR1* | H3K27Ac | ENCFF190JDV | Yes | Yes |
| *MARCHF6* | H3K27Ac | ENCFF190JDV | Yes | Yes |
| *TAB2* | H3K27Ac | ENCFF190JDV | Yes | Yes |
| *ELAVL2* | H3K27Ac | ENCFF190JDV | Yes | Yes |
| *KMT5B* | H3K27Ac | ENCFF190JDV | Yes | Yes |
| *MED13L* | H3K27Ac | ENCFF190JDV | Yes | Yes |
| *ITFG1* | H3K27Ac | ENCFF190JDV | Yes | Yes |

H3K4me3

ENCSR000AOU <https://www.encodeproject.org/experiments/ENCSR000AOU/>, Homo sapiens astrocyte

H3K27Ac

ENCSR000AOQ <https://www.encodeproject.org/experiments/ENCSR000AOQ/>, Homo sapiens astrocyte
