# Supplementary material for: FOXR2 activation is not exclusive of CNS neuroblastoma
Source: Neuro Oncol. 2025 Apr 15;27(7):1801–12. doi: 10.1093/neuonc/noaf076 (PMC12417820; doi:10.1093/neuonc/noaf076)
Supplement: noaf076_suppl_Supplementary_Tables_1-7_Figures_1-9 [file noaf076_suppl_supplementary_tables_1-7_figures_1-9.zip › Supplementary Legends_20250106.docx]

**Supplementary Legends**

**Supplementary Figure 1 Graphic overview of clinical characteristics**

**Supplementary Figure 2 Event-free and overall survival of histone H3 K27M-mutant diffuse midline glioma with and without *FOXR2* activation**

**Supplementary Figure 3 *FOXR2* coverage**

RNA coverage tracks for several *FOXR2*-activated and non-activated samples visualized in IGV^1^. In activated samples, there are peaks in coverage at activated *FOXR2* exons, and there is generally coverage throughout *FOXR2* exon 1 and upstream, depending on the type of activation. Non-activated samples do not have peaks in coverage at annotated *FOXR2* exons and instead have sporadic and low coverage across the region. Samples with *FOXR2* SV events have different DNA breakpoints upstream of the *FOXR2* exon with the fusion event in RNA. The DNA breakpoint is generally indicated where RNA coverage begins. DNA and RNA breakpoints are indicated with red and magenta arrows, respectively. The DNA breakpoint for SJST032384_D1 was not found (no WGS data available for this sample and no intronic RNA soft clips) and SJBT031704_D1 was beyond the limits of the figure (chrX:55,534,637).

**Supplementary Figure 4 Promoter activation events**

RNA bam alignment of cohort samples SJHGG140_D (top, activation of exon -7) and SJHGG112_D (bottom, activation of exon -3) visualized in IGV. The coverage track shows RNA sequencing coverage across the *FOXR2* locus and coverage peaks at activated *FOXR2* exons. The junctions track indicates the presence and frequency of split (spliced) read blocks. The bam track shows individual reads (gray), with blue connecting lines indicating split (spliced) reads. Spliced reads support the presence of the annotated *FOXR2* transcripts shown at the bottom of the plot.

**Supplementary Figure 5 SV events**

RNA bam alignment of cohort samples SJBT032905_D1 (top, SV exon -6), SJMB034234_D1 (middle, SV exon -2), and SJHGG013_D (bottom, SV exon 1) visualized in IGV. Tracks are as described in Supplementary Figure 3.

**Supplementary Figure 6 FOXR2 partners**

**Supplementary Figure 7 LINE-1 insertion**

RNA bam alignment of cohort samples SJHGG030242_D1 (top) and SJBT032161_D1 (bottom) visualized in IGV. Tracks are as described in Supplementary Figure 3. SJHGG030242_D1 was previously described as a LINE-1 insertion event^2^. Identical soft clipped reads at the 5’ end of *FOXR2* exon -2 in SJBT032161_D1 indicate that *FOXR2* activation is also a result of a LINE-1 insertion event in this case. The soft clipped reads blat to many locations across the genome corresponding to LINE-1 sites.

**Supplementary Figure 8 CpG methylation levels (beta values) of the *FOXR2* and adjacent loci**

*FOXR2* CpG sites cg09113017, cg17779979, and cg26217484 showed significant hypermethylation in *FOXR2*-activated CNS tumors.

**Supplementary Figure 9 Expressions patterns of genes that donated their promoters**

**Supplementary Table 1 Molecular information**

**Supplementary Table 2 Baseline characteristics of the study cohort**

**Supplementary Table 3 Characteristics of DMGs with and without *FOXR2* activation**

**Supplemental Table 4 Key baseline imaging features of *FOXR2*-activated CNS tumors**

**Supplementary Table 5 The presence of H3K27ac and H3K4me3 mark peaks within 25kb of the structural variation breakpoints among the *FOXR2* partner genes in normal brain samples according to Encode, suggesting the association with enhancers or promoters**

**Supplementary Table 6 Overview of *FOXR2* partner genes**

**Supplementary Table 7 Gene set enrichment analysis identified enrichment of specific upstream transcription factors of genes in the differentially methylated regions in *FOXR2*-activated CNS tumors**

**References**

**1.** Robinson JT, Thorvaldsdottir H, Winckler W, et al. Integrative genomics viewer. *Nat Biotechnol.* 2011; 29(1):24-26.

**2.** Flasch DA, Chen X, Ju B, et al. Somatic LINE-1 promoter acquisition drives oncogenic FOXR2 activation in pediatric brain tumor. *Acta Neuropathol.* 2022; 143(5):605-607.
