# Supplementary figures and images for: FOXR2 activation is not exclusive of CNS neuroblastoma
Source: Neuro Oncol. 2025 Apr 15;27(7):1801–12. doi: 10.1093/neuonc/noaf076 (PMC12417820; doi:10.1093/neuonc/noaf076)

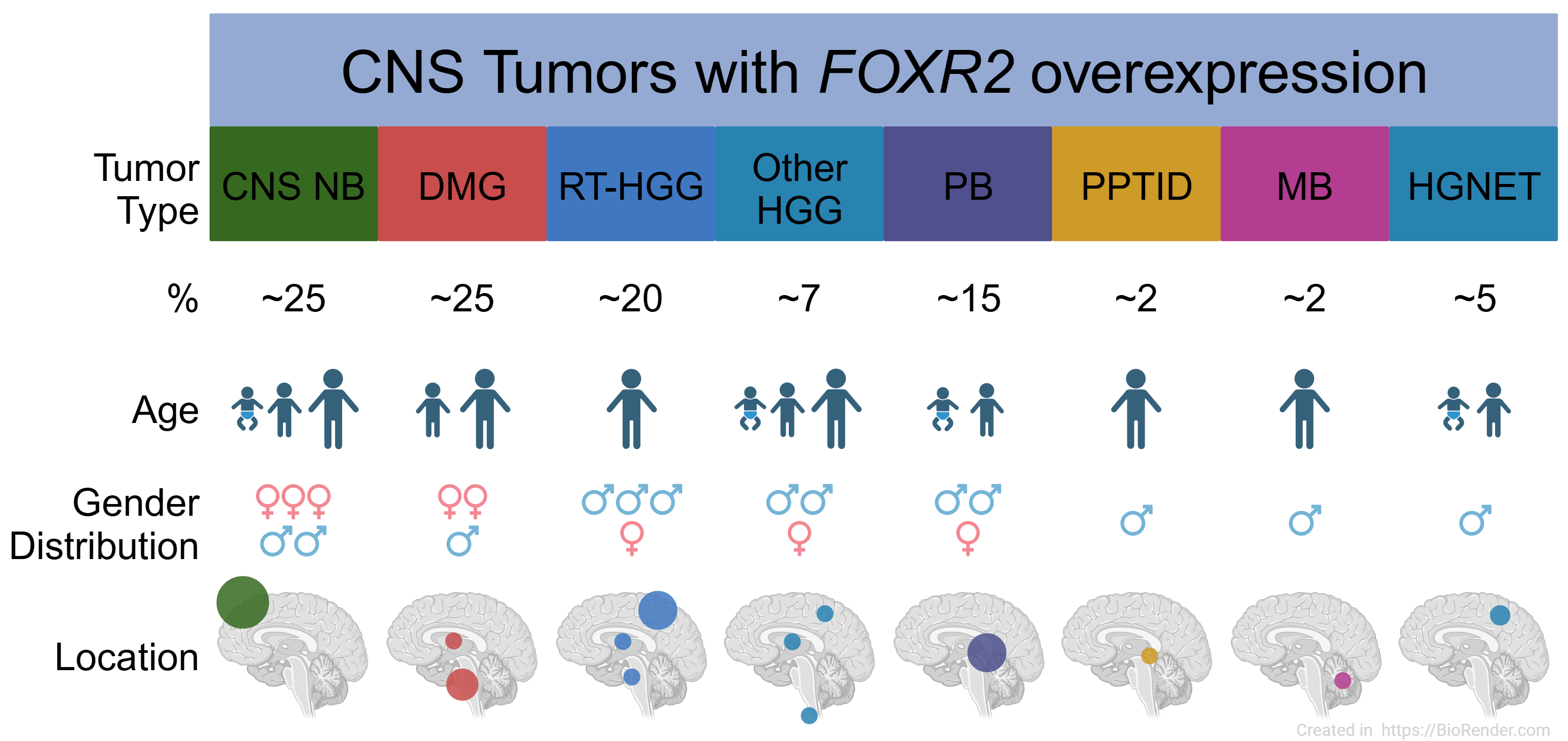

Supplement: noaf076_suppl_Supplementary_Tables_1-7_Figures_1-9 [file noaf076_suppl_supplementary_tables_1-7_figures_1-9.zip › Suppl Fig. 1_Graphic overview_20250225.tif]

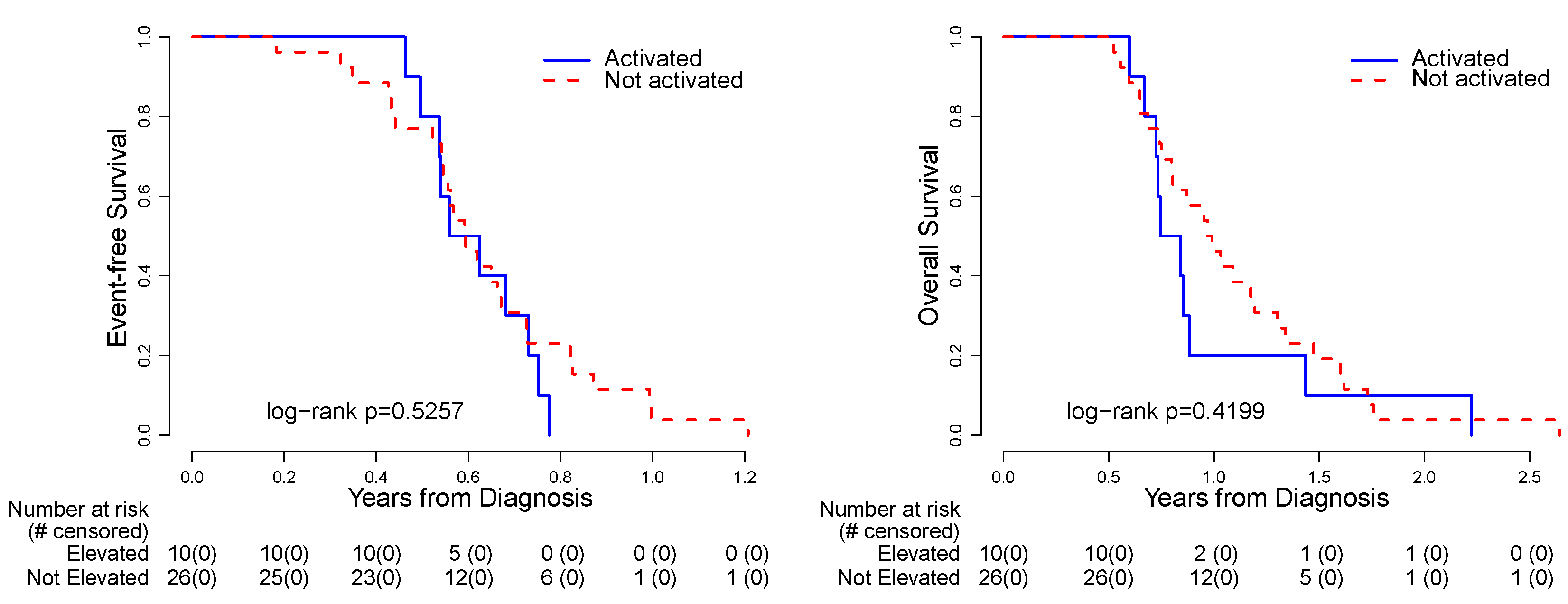

Supplement: noaf076_suppl_Supplementary_Tables_1-7_Figures_1-9 [file noaf076_suppl_supplementary_tables_1-7_figures_1-9.zip › Suppl Fig. 2_FOXR2 DMG PFS & OS_20250127.tif]

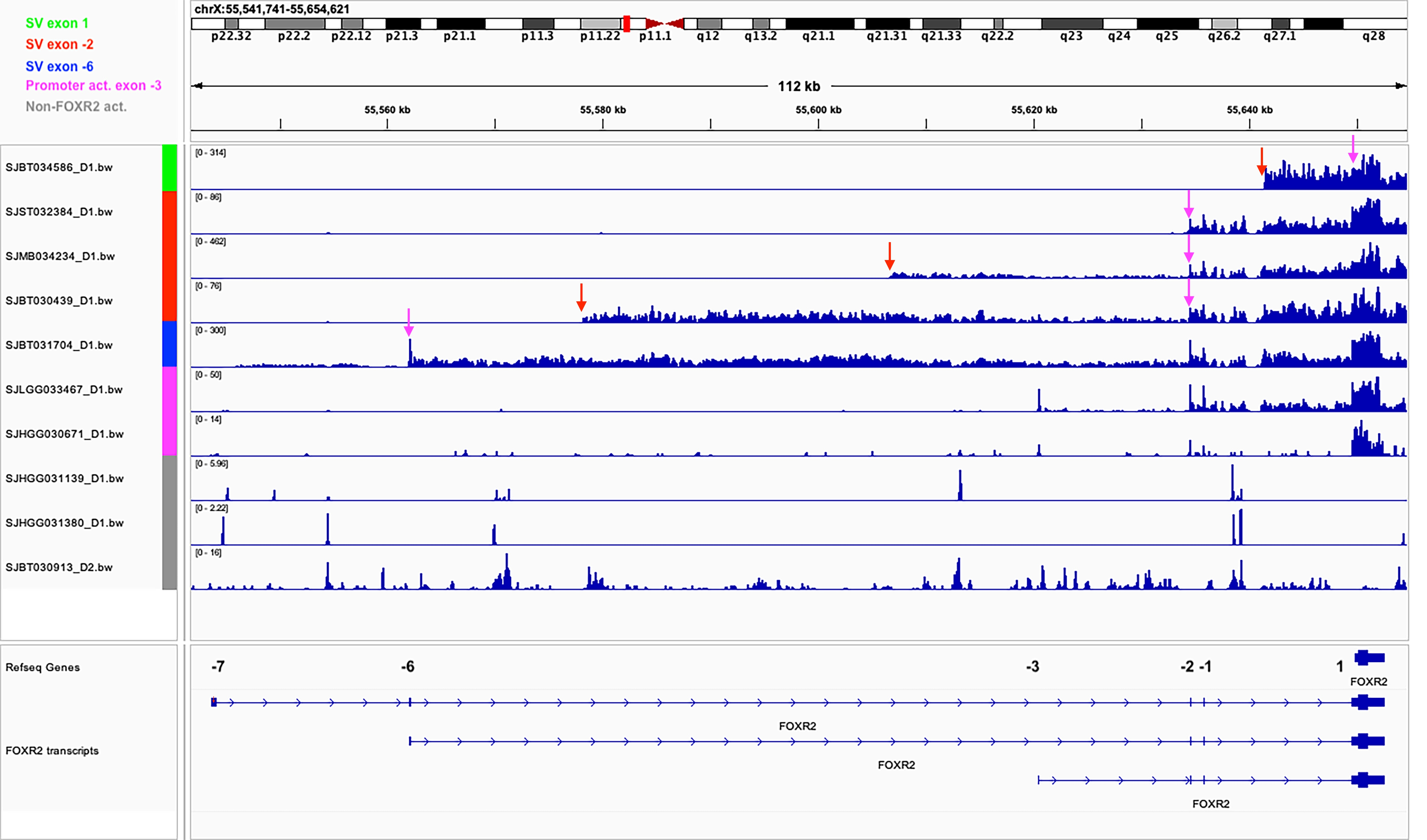

Supplement: noaf076_suppl_Supplementary_Tables_1-7_Figures_1-9 [file noaf076_suppl_supplementary_tables_1-7_figures_1-9.zip › Suppl Fig. 3_FOXR2 coverage_20250127.tif]

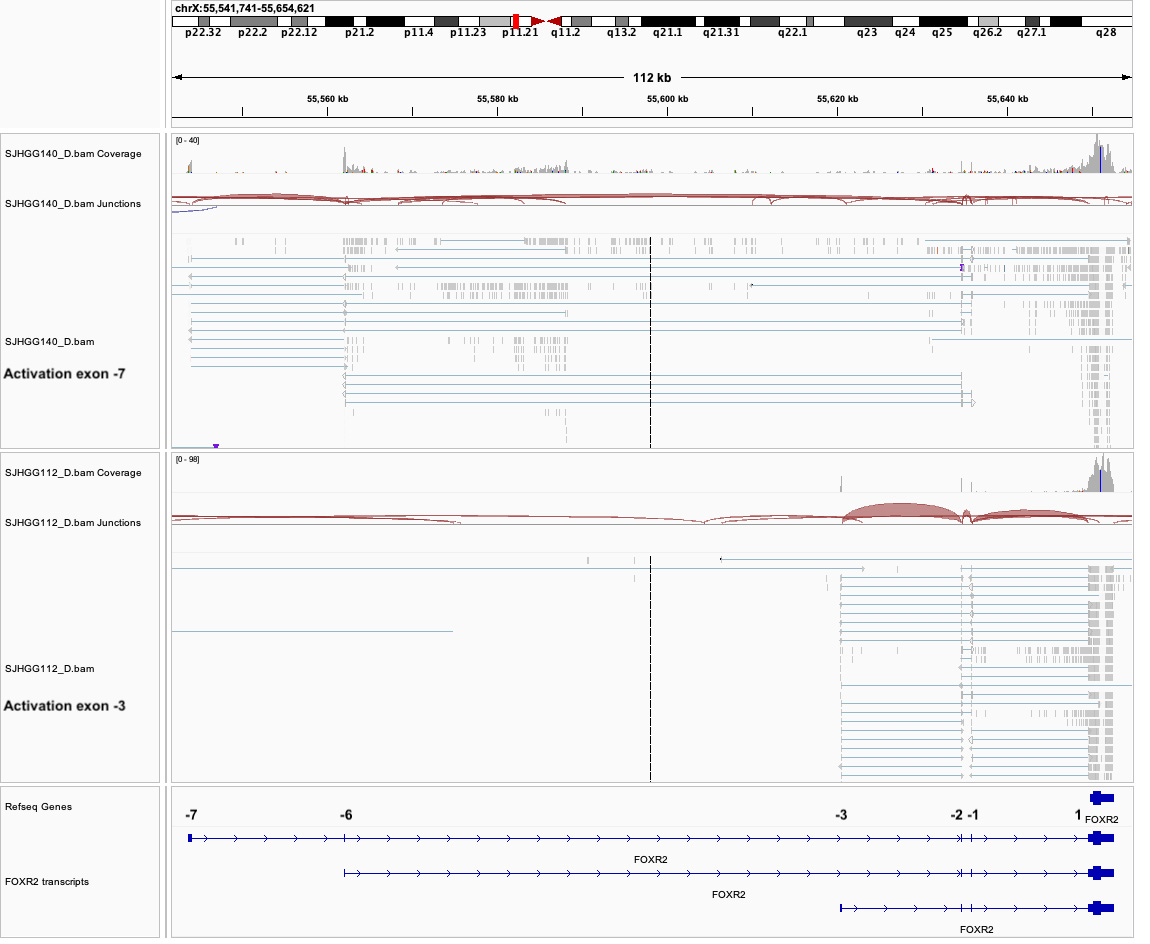

Supplement: noaf076_suppl_Supplementary_Tables_1-7_Figures_1-9 [file noaf076_suppl_supplementary_tables_1-7_figures_1-9.zip › Suppl Fig. 4_Promoter activation events_20250127.tif]

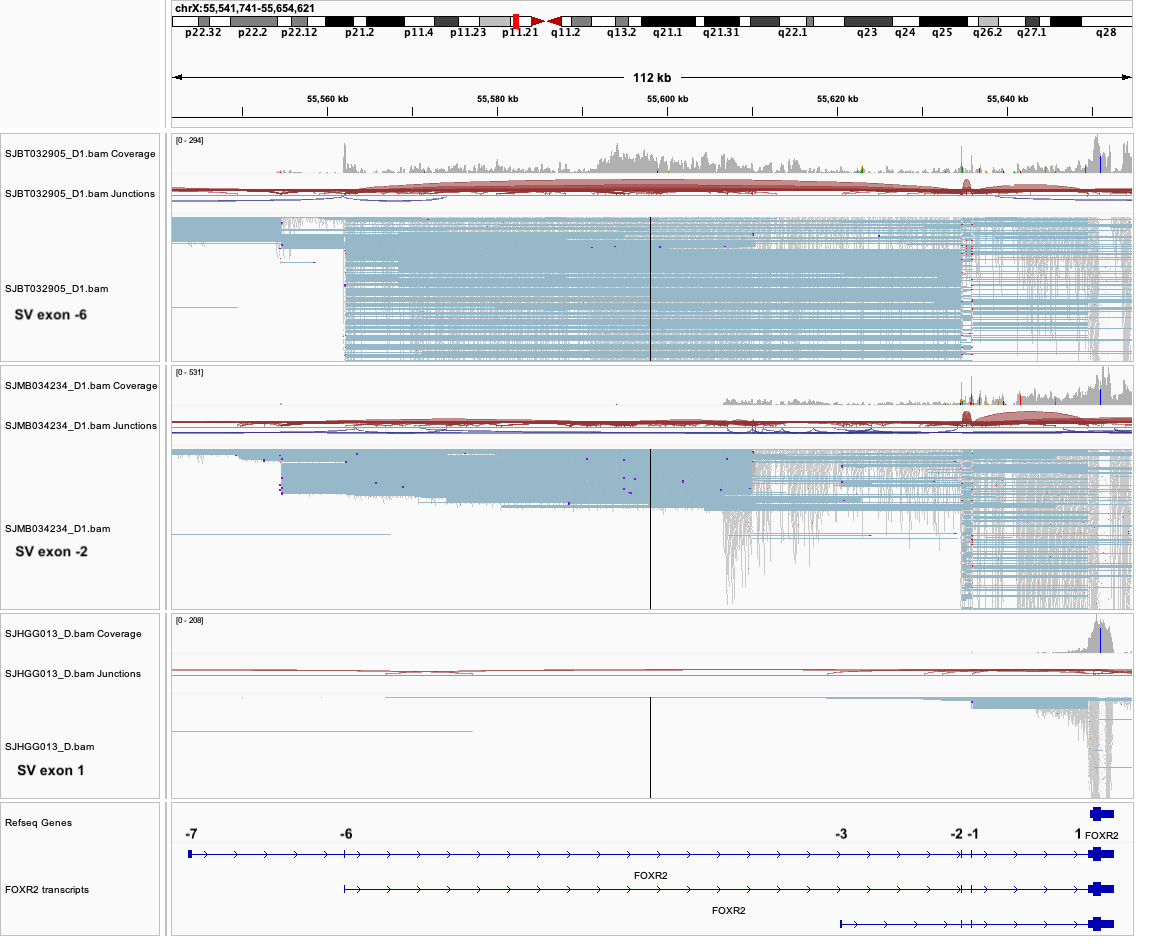

Supplement: noaf076_suppl_Supplementary_Tables_1-7_Figures_1-9 [file noaf076_suppl_supplementary_tables_1-7_figures_1-9.zip › Suppl Fig. 5_SV events_20250127.tif]

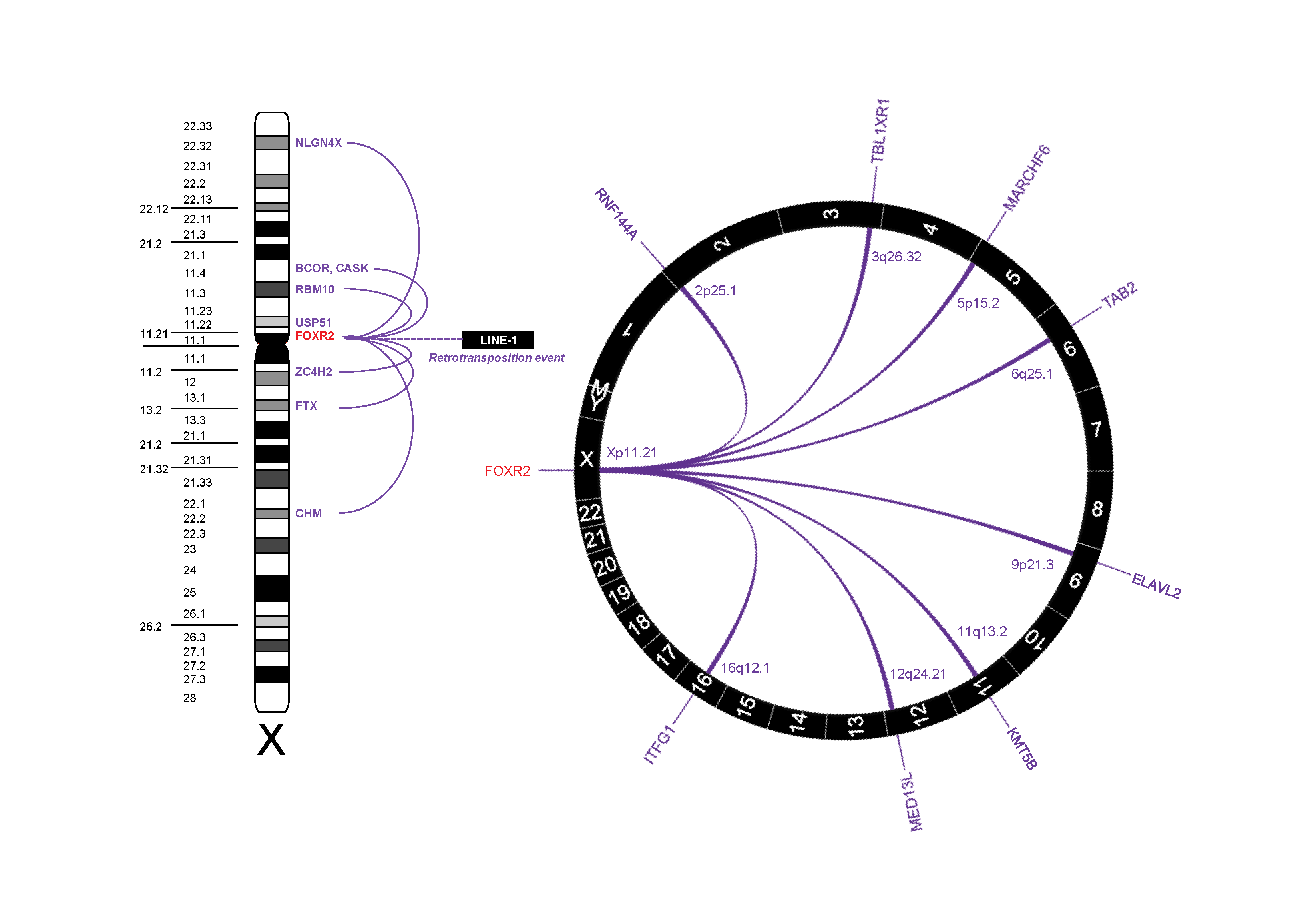

Supplement: noaf076_suppl_Supplementary_Tables_1-7_Figures_1-9 [file noaf076_suppl_supplementary_tables_1-7_figures_1-9.zip › Suppl Fig. 6_FOXR2 partner chr_20250127.tif]

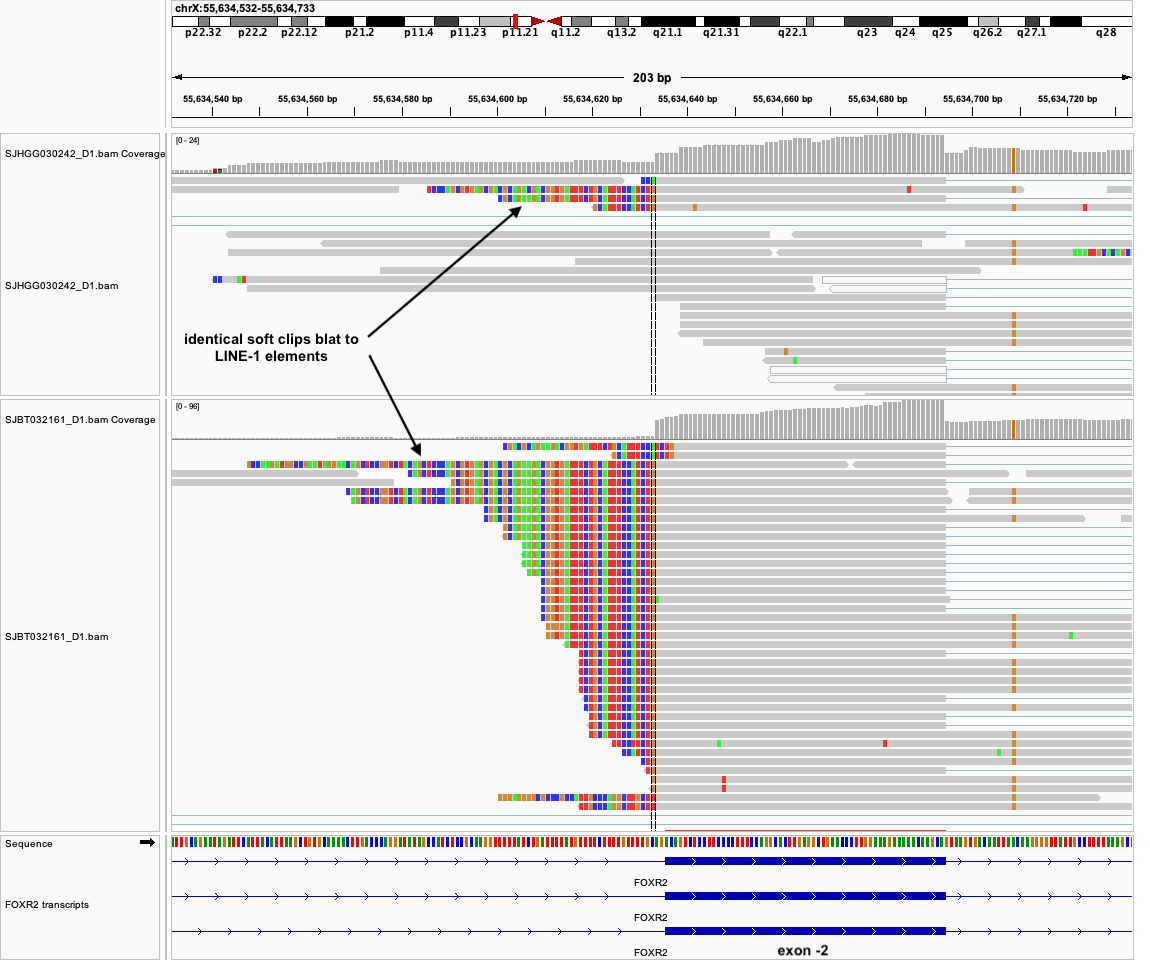

Supplement: noaf076_suppl_Supplementary_Tables_1-7_Figures_1-9 [file noaf076_suppl_supplementary_tables_1-7_figures_1-9.zip › Suppl Fig. 7_LINE-1 insertion_20250127.tif]

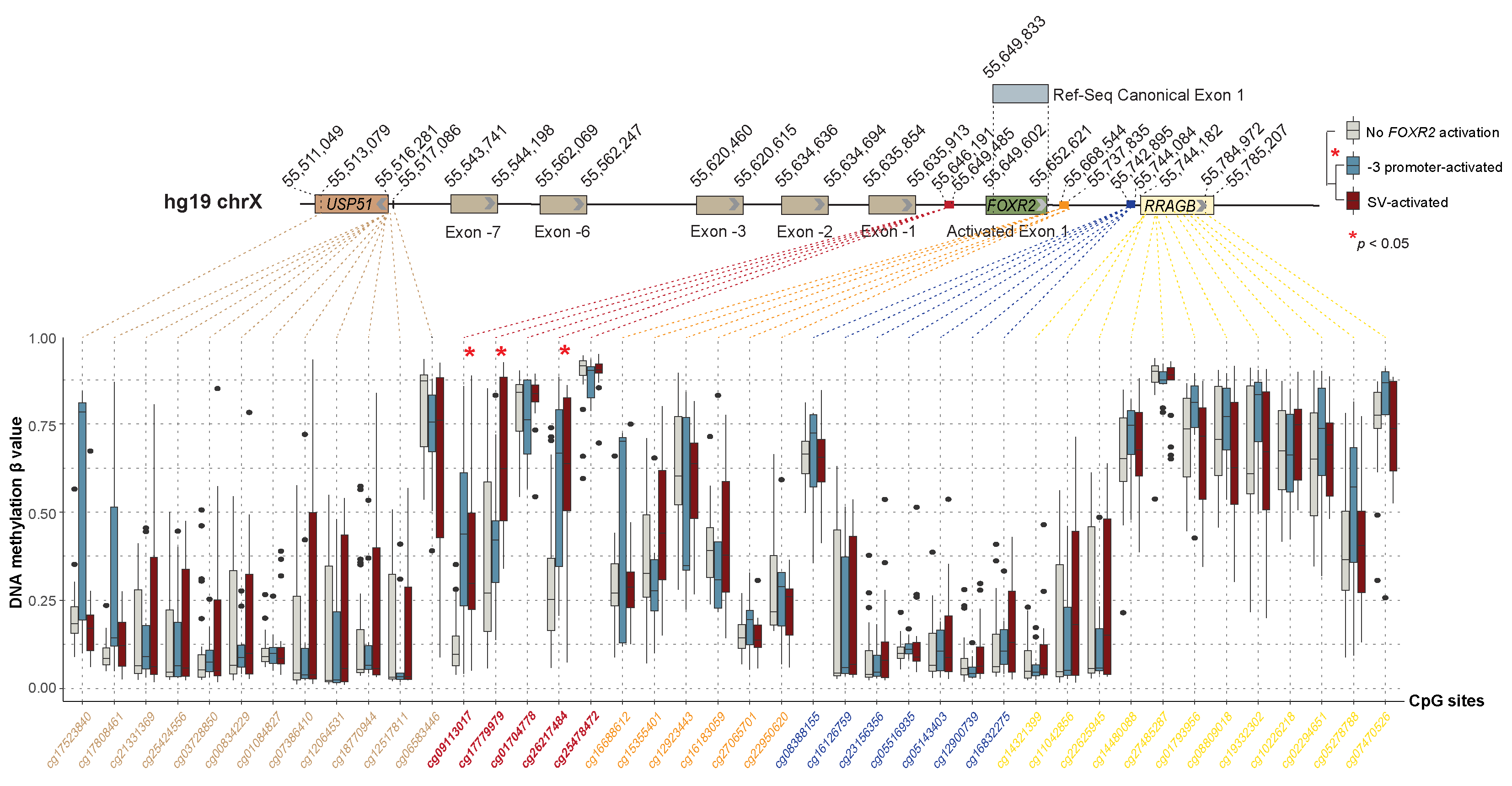

Supplement: noaf076_suppl_Supplementary_Tables_1-7_Figures_1-9 [file noaf076_suppl_supplementary_tables_1-7_figures_1-9.zip › Suppl Fig. 8_FOXR2 CpG methylation_20250127.tif]

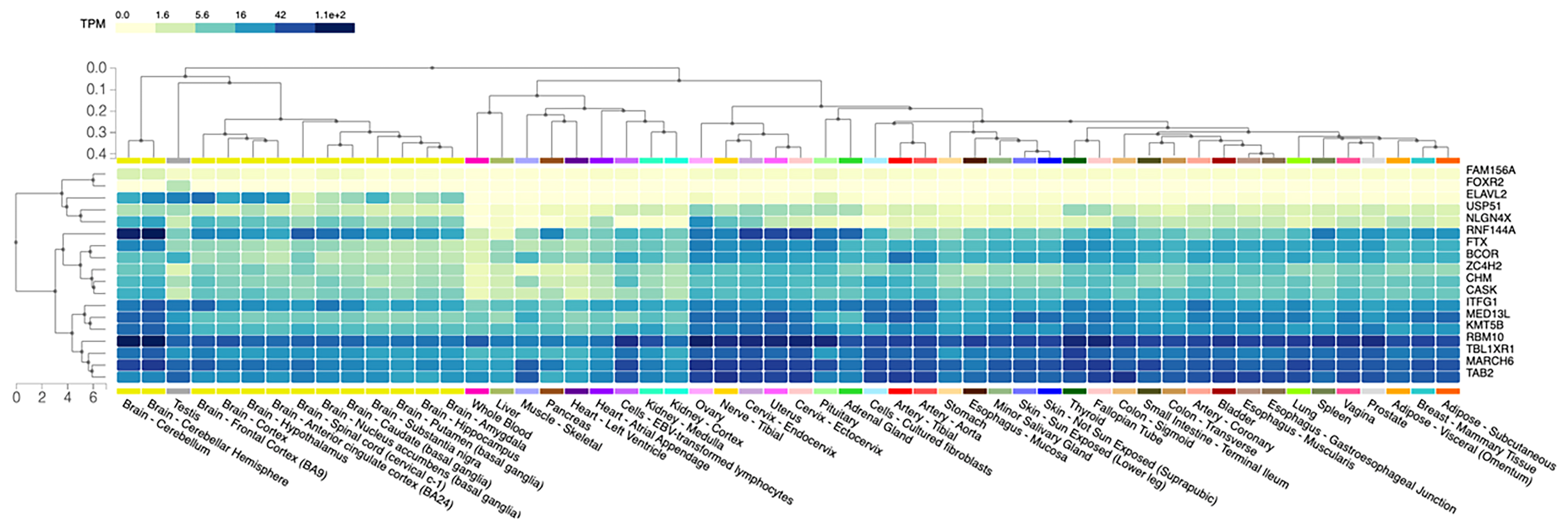

Supplement: noaf076_suppl_Supplementary_Tables_1-7_Figures_1-9 [file noaf076_suppl_supplementary_tables_1-7_figures_1-9.zip › Suppl Fig. 9_GTEx_20250127.tif]
